# Body composition measures as a determinant of Alpelisib related toxicity

**DOI:** 10.1007/s10549-024-07315-9

**Published:** 2024-04-07

**Authors:** Eliya Shachar, Ari Raphael, Uriel Katz, Rivka Kessner, Shlomit Strulov Shachar

**Affiliations:** 1 Oncology Department, Tel Aviv Sourasky Medical Center, 6 Weizmann Street, Tel Aviv, Israel; 2 Faculty of Medicine, Tel Aviv University, Tel Aviv, Israel; 3 Radiology Department, Tel Aviv Sourasky Medical Center, Tel Aviv, Israel

**Keywords:** Metastatic breast cancer, Sarcopenia, Muscle attenuation, Skeletal muscle index, Skeletal muscle gauge, Toxicity, Survival, Alpelisib, Hyperglycemia, Adipose tissue

## Abstract

**Background:**

Body composition has emerged as an important prognostic factor in patients treated with cancer. Severe depletion of skeletal muscle, sarcopenia, has been associated with poor performance status and worse oncological outcomes. We studied patients with metastatic breast cancer receiving alpelisib, to determine if sarcopenia and additional body composition measures accounting for muscle and adiposity are associated with toxicity.

**Methods:**

A retrospective observational analysis was conducted, including 38 women with metastatic breast cancer and a PIK3CA mutation, treated with alpelisib as advanced line of therapy. Sarcopenia was determined by measuring skeletal muscle cross-sectional area at the third lumbar vertebra using computerized tomography. Various body composition metrics were assessed along with drug toxicity, dose reductions, treatment discontinuation, hospitalizations, time to treatment failure and overall survival.

**Results:**

Sarcopenia was observed in half of the patients (n = 19, 50%), spanning normal weight, overweight, and obese individuals. Among the body composition measures, lower skeletal muscle density (SMD) was associated with an increased risk of treatment-related hyperglycaemia (P = 0.03). Additionally, lower visceral adipose tissue (VAT) was associated with alpelisib-induced rash (P = 0.04) and hospitalizations (P = 0.04). Notably, alpelisib treatment discontinuation was not impacted by alpelisib toxicity.

**Conclusion:**

Body composition measures, specifically SMD and VAT may provide an opportunity to identify patients at higher risk for severe alpelisib related hyperglycemia, and cutaneous toxicity. These findings suggest the potential use of body composition assessment to caution toxicity risk, allowing for personalized therapeutic observation and intervention.

**Supplementary Information:**

The online version contains supplementary material available at 10.1007/s10549-024-07315-9.

## Introduction

Breast cancer is the most commonly diagnosed malignancy among women in the United States, excluding nonmelanoma of the skin, and the second leading cause of cancer death in women, after lung cancer [1]. Hormone receptor positive (HR +), human epidermal growth factor receptor‐2–negative (HER2–) breast cancer subtype, comprises more than 70% of metastatic breast cancers (MBC) [2, 3]. The 5-year relative survival of patients diagnosed with metastatic disease from 2012 to 2018 was 29% [1]. In the United States in 2023, there were 297,790 new cases of female breast cancer, and 43,170 estimated deaths [4].

First‐line treatment of patients with HR + HER2– MBC, includes endocrine therapy (ET) combined with a cyclin‐dependent kinase 4/6 inhibitor (CDK4/6i). However, acquired resistance to ET presents a great challenge [5].

Forty-percent of patients with HR + HER2- breast cancer harbor activating mutations in the PIK3CA gene, inducing hyperactivation of the alpha-isoform (p110α) of phosphatidylinositol 3-kinase (PI3K) [6]. Alpelisib is an oral small-molecule, α-specific PI3K inhibitor, which selectively inhibits the p110α with greater efficacy than other isoforms [7].

The SOLAR1 phase 3 randomized double-blind trial led to the FDA approval of alpelisib and fulvestrant, demonstrating prolonged progression-free survival (PFS) among patients with PIK3CA-mutated HR + HER2- MBC, who had received previous endocrine therapy [8, 9]. The estimated median PFS in the alpelisib plus fulvestrant arm was 11 months compared with 5.7 months in the placebo plus fulvestrant arm (HR, 0.65; 95% CI, 0.50–0.85; *P* = 0.001). Nevertheless, alpelisib is associated with frequent adverse events of any grade among patients; high rates of adverse reactions were reported among patients in the SOLAR1 trial, including hyperglycemia (63.7%), diarrhea (57.7%), nausea (44.7%), decreased appetite (35.6%), and rash (35.6%). Alpelisib is given at a fixed dose (300 mg daily) regardless of variables such as adiposity, muscle mass, and sarcopenia.

Poor body composition metrics (BCM) have been associated with inferior oncological outcomes in breast cancer, worse survival, reduced chemotherapy adherence, and increased odds of experiencing chemotherapy and endocrine-related side effects in patients with breast cancer [10–12].

More research is necessary examining the potential use of BCM to predict treatment toxicity and outcomes among various antineoplastic therapies.

Although there have been various works evaluating BCM among breast cancer patients, none have addressed alpelisib or the PI3K inhibitor drug class. We investigated the association of BCM, including muscle and adipose tissue, with drug adverse events (AE) among patients treated with alpelisib and PIK3CA mutated HR + HER2- MBC.

## Methods

### Participants

This single center retrospective analysis included patients with HR + HER2- MBC harboring a mutation in the PIK3CA gene and treated with alpelisib at Tel Aviv Medical Center (TAMC) between October 2015 and July 2023. Eligible patients were females, 21 years of age and older, Eastern cooperative Oncology Group performance status (ECOG PS) 0–3 [13], with a baseline abdominal CT scan dating no more than 30 days prior to therapy initiation, digital images available for muscle mass assessment, and complete electronic medical records. Patient data was extracted and collected from the institutional electronic database. The study was approved by the TAMC Institutional Review Board (Helsinki ethics approval number 0611-21-TLV).

### Toxicity grading measures

Patient demographics and AE were extracted from the electronic medical records. Grading severity was scaled according to the toxicity grades 1–5 of National Cancer Institute Common Toxicity Criteria for adverse events (NCI- CTCAE, Version 4.03) [14]. We limited our review of adverse effects based on the commonly reported events in the literature including hyperglycemia, rash, gastrointestinal toxicity (diarrhea, nausea, abdominal pain, stomatitis, vomiting), neurotoxicity, dose reductions, treatment delays, hospitalizations due to treatment toxicity, and death. We measured an additional parameter, “Severe toxicity”, a subset of patients who suffered from heightened toxicity, including dose reductions, treatment delays, toxicity grade ≥ 3, and hospitalizations.

### Body composition analysis

Measures of body composition were evaluated including body surface area (BSA), and body mass index (BMI). BMI was calculated using the following formula: BMI = weight (kg)/height^2^ (m^2^) [15, 16]. Obese was classified as patients with a BMI ≥ 30.0 kg/m^2^. BSA was calculated using the Mosteller formula: BSA (m^2^) = $\sqrt{\left[\frac{\mathrm{height }\left({\text{cm}}\right) \times \mathrm{ weight }\left({\text{kg}}\right)}{3600}\right]}$ [17].

#### CT-computed body composition measures

Abdominal CT images were acquired from the TAMC Picture Archiving and Communication System (Philips Algotec, Ra’anana, Israel) and analyses were conducted with the guidance of a radiologist. Axial plane CT images at the level of third lumbar vertebrae (L3) were evaluated. L3 lumbar segments were processed using automated image segmentation software sliceOmatic (Tomovision, Montreal, Canada) [18, 19]. The software recognizes muscle tissue based on density threshold between − 29 and + 150 Housfield units (HU), while using a priori information about the L3 muscle shape to avoid mislabeling parts of the neighboring organs that also have HU values in the − 29 + 150 range. Cross-sectional areas (cm^2^) of the sum of all L3 regional muscles (psoas, paraspinal, and abdominal wall muscles) were computed for each image, and the average value for the two images was calculated for each patient. The program provides a highly accurate estimation of the cross-sectional lean tissue area and skeletal muscle area [20–23].

Sarcopenia, a decrease in skeletal muscle index (in women < 38cm^2^/m^2^), was previously defined in an Asian population using reported cut-off values [24]. These values were chosen as they have been extensively investigated, and examined in the first prospective trial of Israeli patients, while other cutoffs have been reflective of Western populations [25]. For women of the study population, sarcopenia was defined by skeletal muscle index (SMI). SMI was calculated using the following formula: (L3-muscle area-cm^2^)/(patient height-m^2^). An SMI of < 38 cm^2^/m^2^ was considered sarcopenic, based on previously derived optimal stratification statistics, correlating SMI with worse prognosis in a population of patients with lung cancer [24]. Estimation of lean body mass (LBM) was calculated using the formula described by Mourtzakis et al. (LBM (kg) = [(L3 muscle measured by CT (cm^2^) × 0.3) + 6.06]) [26, 27].

Mean skeletal muscle density (SMD) was derived by averaging Hounsfield Units (HU) of skeletal muscle at the level of L3 vertebrae. The attenuation measurement of skeletal muscle is used as a non-invasive radiological technique to indirectly assess muscle fat content. The density of skeletal muscle is inversely related to muscle fat content [28]. Since SMI and SMD are each significantly associated with outcome [29–31], we explored whether combining the two skeletal muscle measures, may provide a stronger correlation with outcome and toxicity. To integrate both SMI and SMD, we evaluated patient skeletal muscle gauge (SMG), which was calculated by multiplying SMI × SMD, as first presented by Weinberg et al. [32]. The units for SMG are: (cm^2 tissue * average HU)/(m^2 height) for simplicity we chose to represent them as arbitrary units (AU) [32]. Subcutaneous adipose tissue (SAT) area was calculated from extramuscular tissue with density between − 190 and − 30 HU and visceral adipose tissue (VAT) from non-subcutaneous tissue with density between − 150 and − 50 HU.

### Oncological measures

Furthermore, we also collected additional oncological parameters including patient age at diagnosis with metastatic disease and lines of prior therapies. Time to treatment failure (TTF) and overall survival (OS) were assessed.

### Statistical analysis

Data that met the normal distribution assumptions, confirmed by the Kolmogorov–Smirnov test and histogram underwent parametric testing using the two-group t-test and were presented as mean ± standard deviation. For data that did not adhere to a normal distribution, nonparametric tests were employed, specifically the Mann–Whitney U-test, with results reported as median (IQR), or the Fisher’s exact test when appropriate. A binary logistic regression model was used to estimate the Odds ratio. A P-value of less than 0.05 was deemed statistically significant. All statistical evaluations were conducted using IBM SPSS version 29.0.1.

## Results

### Patient characteristics and body composition

Thirty-eight patients diagnosed with HR + HER2- MBC and a PIK3CA mutation, treated with alpelisib at TAMC between October 2015 and July 2023, met eligibility criteria and were included in the analysis. Patient clinical characteristics, body composition measures and toxicity outcomes are described in Table 1. The median age was 70 years (interquartile range [IQR], 57–78). Approximately half of the women (n = 20, 53%) were treated with alpelisib up to third line or below, 18 (47%) patients received alpelisib as fourth or greater line of treatment, with a median of 3 prior lines of therapy (IQR, 2–4). Patient mean weight was 60 kg (standard deviation [SD] ± 14). Mean BMI was 23.9 ± 5.9 kg/m^2^, and among the study population, 10 (26%) patients were obese. Median BSA was 1.6 m^2^ (IQR, 1.5–1.7).

**Table 1:** Patient characteristics, body composition measures and toxicity outcomes

Variables	N = 38
Age, median (IQR) years	70 (57–78)
Female, n (%)	38 (100)
ECOG n, (%)	
0	8 (32)
1	10 (40)
2	5 (20)
3	2 (8)
Alpelisib treatment line n, (%)	
≤ 3	20 (53)
≥ 4	18 (47)
Alpelisib, median treatment line (IQR)	3 (2–4)
Weight, mean ± SD, kg	60.2 ± 14
BMI, mean ± SD, kg/m^2^	23.9 ± 5.9
BMI category, n (%)	
Healthy weight	22 (58)
Overweight	6 (16)
Obese	10 (26)
BSA median (IQR), m^2^	1.6 (1.5–1.7)
SMI, mean ± SD, cm^2^/m^2^	35.5 ± 11
SMG, median (IQR), AU	1142 (935–1511)
LBM, median (IQR), kg	34.4 (31–37.1)
SMA, mean ± SD, cm^2^	87.9 ± 29.6
SMD, HU	32 (10.5)
Sarcopenic < 38 n (%)	
Yes	19 (50)
Dose reduction	
Yes	16 (42.1)
Dose interruption	
Yes	26 (68.4)
Hospitalizations due to drug, number (%)	
Yes (%)	6 (16)
AE	
Grade ≥ 2 AE	
Yes	31 (81.6)
Grade ≥ 3 AE	
Yes	15 (39.5)

Abbreviations: *IQR* interquartile range, *SD* standard deviation; Plus–minus values are means ± SD; *BMI* body mass index

BMI-Healthy < 25 kg/m^2^; overweight 25–30 kg/m^2^; obese, ≥ 30 kg/m^2^

AE = adverse event, AU = arbitrary units, BSA = body surface area, SMI = skeletal muscle index, Defined as SMI < 38cm^2^/m^2^

SMD- skeletal muscle density was derived by averaging Hounsfield Units (HU) of skeletal muscle at the L3 vertebrae

SMG- skeletal muscle gauge was calculated by multiplying SMI × SMD; the units for SMG are: (cm^2 tissue * average HU)/(m^2 height) for simplicity we chose to represent them as arbitrary units (AU)

LMB- lean body mass (kg) = 0·30 × [skeletal muscle at L3 using CT (cm^2^)] + 6·06]

SMA- skeletal muscle area (cm^2^)

**Fig. 1:**
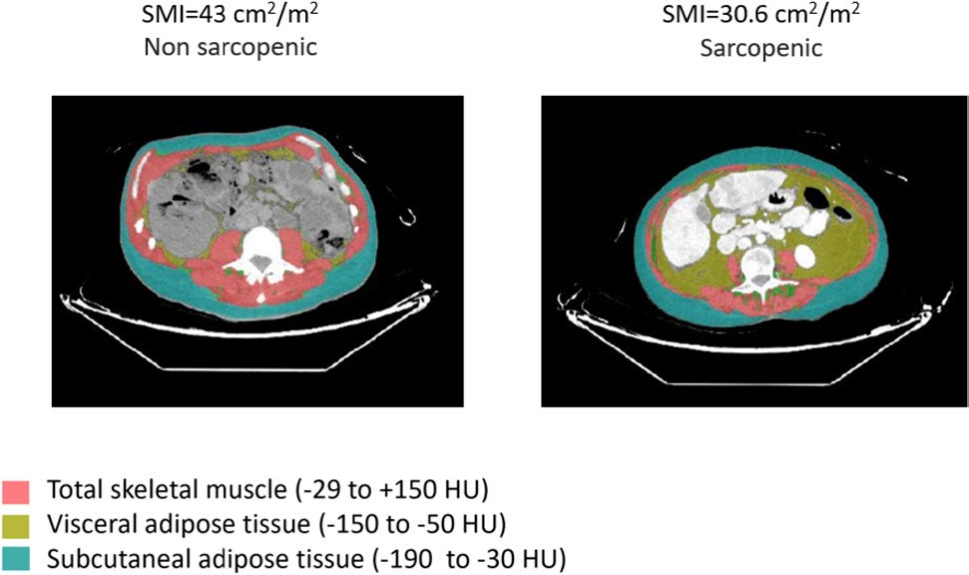
Example of sarcopenia, two patients with metastatic breast cancer, Left, normal SMI (43cm^2^/m^2^) non sarcopenic; Right, low SMI (30.6 cm^2^/m^2^) sarcopenic

CT-based body composition indices were available and calculated for all patients. Patient mean SMI was 35.5 cm^2^/m^2^ as demonstrated in Fig. 1, and a median SMG of 1142 AU (IQR, 935–1511). Patient median LBM was 34.4 kg (IQR, 31–37.1). The mean SMA was 87.9 cm^2^ ± 29.6 [SD]), and SMD was 32 HU.

Half of the patients were sarcopenic, (n = 19, 50%). The study population PIK3CA mutations are presented in Supplementary Table 1.

### Toxicity outcomes

Among the study population, almost half of the patients had a dose reduction of alpelisib (16, 42.1%), and 26 patients (68.4%) a dose interruption of therapy (Table 1).

A minority were hospitalized resulting from toxicity of the treatment (n = 6, 16%). Only 3 (8%) patients did not experience any AE. The majority of patients encountered AE grade ≥ 2 (n = 31, 81.6%) and 15 women (39.5%) suffered from grade ≥ 3 AE.

### Body composition as a predictor of increased Alpelisib toxicity

Among women with grade ≥ 2 AE, age, treatment line, ECOG PS, sarcopenia (SMI < 38, and SMI teritial divisions), SMG, and LBM tertiles did not provide additional measures in determining the likelihood of increased drug toxicity (gastrointestinal, haematological, hyperglycaemia, and rash), hospitalizations, dose reductions and interruptions of alpelisib, as demonstrated in Table 2.

**Table 2:** Association of baseline characteristics, treatment, with adverse events grade ≥ 2

	At lease one AE Grade ≥ 2, n (%)		
	Yes	No	P-value^a^
Age group			P = 0.330
< 60	10 (27)	2 (5.4)	
60–64	2 (5.4)	1 (2.7)	
65–69	2 (5.4)	1 (2.7)	
70–74(n = X)	5 (13.5)	2 (5.4)	
≥ 75(n = X)	12 (32.4)	0 (0)	
Treatment Line			P = 0.761
≤ 3	16 (43.2)	4 (10.8)	
≥ 4	15 (40.5)	2 (5.4)	
ECOG			P = 0.584
0	7 (28)	1 (4)	
1	8 (32)	2 (8)	
2	5 (20)	0 (0)	
≥ 3	1 (4)	1 (4)	
Sarcopenia (SMI < 38), cm^2^/m^2^			P = 0.187
Yes	14 (40)	5 (14.3)	
No	15 (42.9)	1 (2.9)	
SMI^b^ tertile, cm^2^/m^2^			P = 0.852
1 (< 33.8)	9 (25.7)	3 (8.6)	
2 (33.81–39.84)	10 (28.6)	2 (5.7)	
3 (≥ 39.85)	10 (28.6)	1 (2.9)	
SMG^b^ tertile, AU			P = 0.087
1 (≤ 989)	11 (34.4)	0 (0)	
2 (990–1412)	7 (21.9)	4 (12.5)	
3 (≥ 1413)	8 (25)	2 (6.3)	
LBM^b^ tertile, kg			P = 0.854
1 (≤ 32.02)	10 (83.3)	2 (16.7)	
2 32.03–36.66)	10 (76.9)	3 (23.1)	
3 ≥ 36.67)	11 (91.7)	1 (8.3)	

Abbreviations: *SMG* skeletal muscle gauge, *SMI* skeletal muscle index, *AE* adverse event, *AU* arbitrary units, *BSA* body surface area, *SMI* skeletal muscle index, Defined as SMI < 38cm^2^/m^2^. SMD- skeletal muscle density was derived by averaging Hounsfield Units (HU) of skeletal muscle at the L3 vertebrae. SMG- skeletal muscle gauge was calculated by multiplying SMI × SMD, the units for SMG are: (cm^2 tissue * average HU)/(m^2 height) for simplicity we chose to represent them as arbitrary units (AU). LMB- lean body mass (kg) = 0·30 × [skeletal muscle at L3 using CT (cm^2^)] + 6·06]

SMA- skeletal muscle area (cm^2^)

^a^Chi-square test

^b^Defined as tertiles

Patients with a lower tertiles SMG were likely to have increased risk of Severe toxicity, (22.6%, the upper two thirds which were 3.2%, and 6.5%, respectively, P = 0.02).

When evaluating each toxicity independently, age, ECOG, dose reductions and body composition measures (including BMI, BSA, VAT, SAT, SMA, SMI, SMG), they were not associated with increased toxicity from alpelisib (Table 3).

**Table 3:** Body metric parameters predictive of independent toxicities

	Hyperglycaemia G ≥ 1 P-value	Hyperglycaemia G ≥ 2 P-value	Rash G ≥ 1 P-value	Rash G ≥ 2 P-value
Age	0.785	0.105	0.256	0.312
BMI	0.157	0.143	0.170	0.207
BSA	0.400	0.363	0.283	0.058
VAT, cm^2^	0.023*	0.082	0.227	0.102
VAT, HU	0.009*	0.051**	0.020*	0.043*
SAT, cm^2^	0.016*	0.204	0.145	0.164
SAT, HU	0.021*	0.361	0.907	1
SMD, HU	0.015*	0.024*	0.792	0.442
SMA, cm^2^	0.085	0.315	0.135	0.161
IMAT, HU	0.125	0.547	0.868	0.680
SMI, cm^2^/m^2^	0.125		0.058	0.149
SMG, AU	0.404	0.439	0.823	0.527
Dose reduction	0.340	0.153	0.134	0.133
Hospitalizations	0.564	0.303	0.335	0.042*
LBM	0.73	0.165	0.48	0.86

Abbreviations: *VAT* visceral adipose tissue, *SAT* subcutaneous adipose tissue, *IMAT* intramuscular adipose tissue

P-value indicates statistical significance in the comparison of mean body metric compositions between groups that possess these characteristics and those that do not

^*^statistically significant P < 0.05, **marginally significant

Risk of hyperglycemia was associated with lower mean VAT (40 ± 32.8 [SD] cm^2^ vs. 103.3 ± 57.7 [SD] cm^2^, P = 0.023), mean SAT (100.8 ± 74.6 [SD] cm^2^ vs. 183.4 ± 66.5 [SD] cm^2^, P = 0.016), mean SMD (41.6 ± 11.6 [SD] HU vs. 29.6 ± 9.2 [SD] HU, P = 0.015), median VAT density, − 76.36 HU (IQR, − 85.31, − 60.27) vs. − 91 HU(IQR, − 98.79, − 81.14, P = 0.009), and median SAT density (− 83.23 HU (IQR, − 97.78, − 81.28) vs. − 101 HU (IQR, − 105.2, − 94.12), P = 0.021). The risk of hyperglycaemia grade ≥ 1 was not associated with age, BMI, BSA, height, SMI, SMG, and LBM.

Among the body composition measures, mean SMD was associated with grade ≥ 2 hyperglycemia, (38 ± 9.6 [SD] HU vs. 28.9 ± 9.7 [SD] HU, P = 0.024). Median VAT was marginally associated with grade ≥ 2 hyperglycemia, − 79.69 HU (IQR, − 92.9, − 75.26) vs. − 91 HU (IQR, − 98.79, − 83.14), P = 0.05).

Rash grade ≥ 2 was associated with lower median VAT (− 88.35 HU (IQR, − 94.43, − 78.22) vs. − 97.8 HU (IQR, − 172, − 89.85), P = 0.043). While grade ≥ 2 rash, was associated with an increased hospitalization (8% of patients hospitalized with a rash vs. 75.7% of patients who were not hospitalized and without a rash, P = 0.042).

Among patients with Severe toxicity, they had a higher risk of developing grade ≥ 2 hyperglycaemia (OR = 9.58, P = 0.01).

None of the body composition metrics were found to be significantly associated with an increased likelihood of having hematological, and gastrointestinal toxicity.

Among the population of patients with sarcopenia (SMI < 38) who were overweight or obese, 8 (23%) women experienced any toxicity grade ≥ 2, 21% hyperglycemia grade ≥ 2, 16% had a dose reduction or delay, 21% experienced Severe toxicity, while none were hospitalized or experienced rash (grade ≥ 2).

### Oncological outcomes

We performed an analysis of TTF and OS and found that they were not statistically different across patients with various body composition metrics, drug toxicity, and dose reductions.

## Discussion

To our knowledge, this is the first report of the impact of body composition measures on alpelisib toxicity and adherence to therapy. Body composition measures were useful in identifying patients with increased alpelisib induced hyperglycemia and rash. Other AE were not associated with body metrics. This work demonstrates body composition parameters that may be integrated to identify patients with greater likelihood to develop treatment related toxicities beyond the conventional measures of BMI and BSA, and tailor observation.

Hyperglycemia and rash are common AE of alpelisib resulting from inhibition of the PI3K pathway [33]. P110α is involved in glucose metabolism, mediating the response to insulin in skeletal muscle, liver, and fat. PI3K inhibition leads to insulin resistance, interrupting glucose uptake in muscle and adipose tissue, thereby activating hepatic glycogenolysis, resulting in hyperglycemia and compensatory increase in insulin.

Among the body composition measures, mean skeletal muscle density (SMD) was associated with grade ≥ 2 hyperglycemia, thus women with lower SMD were at increased risk of developing treatment induced hyperglycemia.

Previous studies have demonstrated that low SMD is a poor prognostic factor for patients with metastatic pancreatic adenocarcinoma, receiving palliative first line gemcitabine-based chemotherapy [34–36]. Furthermore, patients with pancreatic adenocarcinoma and grade ≥ 3 toxicity was more frequently observed in patients with low SMD. Low SMD indicates intramuscular adipose tissue infiltration and poor muscle strength [37]. Several factors have been implicated to play a role in the onset and progression of sarcopenia. It has been suggested that oxidative stress, chronic inflammation, and mitochondrial dysfunction are involved in muscle atrophy [38]. These factors are thought to influence the balance between protein synthesis and breakdown, inducing apoptosis, leading to pathological loss of significant muscle mass including fiber atrophy, loss and eventually sarcopenia [39]. Oxidative stress, caused by increased reactive oxygen species (ROS) and decreased antioxidant effects, are implicated in disease [40]. Muscle cells produce ROS as a by-product of normal metabolism and are subsequently more susceptible to oxidative stress. Interestingly, hyperglycemia in type 2 diabetes triggers increased production of ROS [41]. ROS activate the ubiquitin–proteasome system and accelerates the degradation of muscle proteins, leading to sarcopenia. Oxidative stress inhibits the Akt/mTOR pathway and the downstream targets, subsequently inhibiting protein synthesis and promoting muscle atrophy.

Additionally, in our work there was a trend seen among women with lower mean visceral adipose tissue (VAT), who were more likely to develop grade ≥ 2 hyperglycemia with alpelisib.

These measures, SMD and VAT may identify a patient population necessitating a more tailored treatment approach and observation, managing glucose control at lower grades and possibly earlier intervention.

Women treated with alpelisib who developed a rash grade ≥ 2, had lower mean visceral adipose tissue, and greater likelihood of hospitalizations. The etiology of alpelisib induced rash is not clear; the maculopapular rash is associated with increased blood eosinophils [42]. Additional research is necessary to identify the mechanism in which inhibition of PI3Kα alters immune cell signaling and results in clinical manifestations of dermatological adverse effects.

Interestingly, half of the study population were deemed sarcopenic, irrespective of BMI.

Additionally, we did not find patient BMI, those who were overweight or obese, or age to be risk factors for treatment toxicity.

TTF and OS were not statistically different across various body composition metrics, drug toxicity, and dose reductions. Notably, patients received alpelisib at various lines of treatment, potentially obscuring the analysis.

Limitation of this study stem from the study design, a retrospective observational analysis of a small heterogeneous population, which may influence the external validity of the results. Additional, alpelisib was administered as an advanced line of therapy, 47% were treated as fourth or greater line, limiting the analysis of time to treatment failure and overall survival.

The appropriate SMI cut-off in the Israeli population has not been thoroughly established. Some definitions of SMI take into account BMI. Thus, the BMI of the patient population, which is impacted by factors such as ethnicity and diet, is relevant to the rate of the observed sarcopenia. The Israeli patient population is influenced by a Mediterranean diet and diverse ethnicities. The first and only Israeli prospective study [43] evaluated various SMI cut-offs including Martin et al. reflective of a Western population and Kimura et al., an Asian population, and demonstrated a statistically significant association between sarcopenia as defined by Kimura et al. and low skeletal muscle gauge with the presence of grade 2 or higher AEs [24, 25].

Given the variability in the correlations between BMI and clinical outcomes in patients with breast cancer, assessment of body composition through distinct body compartments, such as skeletal muscle, and adiposity, separately has evolved as a potentially more informative approach.

Our findings suggest that among the toxicities of alpelisib, hyperglycemia and rash were associated with lower SMD and VAT. These results raise the option to identify patients at higher risk for severe side effects, potentially guiding a more personalized approach for these patients. Future prospective studies are necessary to validate body composition measures as potential predictors of treatment toxicity and develop optimal interventions to mitigate toxicity for this risk group.

### Supplementary Information

Below is the link to the electronic supplementary material.

Supplementary file1 (DOCX 15 KB)

## Data Availability

No datasets were generated or analysed during the current study.
