# Supplementary material for: Body composition measures as a determinant of Alpelisib related toxicity
Source: Breast Cancer Res Treat. 2024 Apr 7;206(2):369–76. doi: 10.1007/s10549-024-07315-9 (PMC11182811; doi:10.1007/s10549-024-07315-9)
Supplement: Supplementary file 1 — Supplementary file1 (DOCX 15 KB) [file 10549_2024_7315_MOESM1_ESM.docx]

**Supplementary**

**Table 1S. Patients PIK3CA mutations**

| **PIK3CA mutation** | **N, (%)** |
| --- | --- |
| Gly106Asp (G106D) | 1 (2.6) |
| Glu110del | 1 (2.6) |
| Asn345Lys (N345K) | 2 (5.3) |
| Glu418Lys (E418K) | 1 (2.6) |
| Pro539Arg (P539R) | 1 (2.6) |
| Glu542Ala (E542A) | 2 (5.3) |
| Glu542Lys (E542K) | 1 (2.6) |
| Glu545Lys (E545K) | 6 (15.8) |
| Glu726Lys (E726K) | 2 (5.3) |
| Thr1025Ala (T1025A) | 1 (2.6) |
| Met1043Ile (M1043I) | 1 (2.6) |
| His1047Arg (H1047R) | 9 (23.7) |
| His1047Leu (H1047L) | 1 (2.6) |
| **Additional PK3CA mutations (double mutations)** | 8 (21.1) |
| Asn107Thr (N107T) | 1 (2.6) |
| Glu546Leu (Q546L) | 1 (2.6) |
| Asp1017His (D1017H) | 1 (2.6) |
| His1047Arg (H1047R) | 5 (13.2) |
